# Development of Hydrophobic Coal-Fly-Ash-Based Ceramic Membrane for Vacuum Membrane Distillation

**DOI:** 10.3390/ma16083153

**Published:** 2023-04-17

**Authors:** Zheng Zhang, Jihao Yang, Run Qi, Jiguang Huang, Haiping Chen, Heng Zhang

**Affiliations:** 1 School of Energy, Power and Mechanical Engineering, North China Electric Power University, Beijing 102206, China; 120202202122@ncepu.edu.cn (Z.Z.); 120212202106@ncepu.edu.cn (J.Y.); qirun@ncepu.edu.cn (R.Q.); ncepuhjg@ncepu.edu.cn (J.H.); hdchp@ncepu.edu.cn (H.C.); 2 Beijing Key Laboratory of Pollutant Monitoring and Control in Thermoelectric Production Process, North China Electric Power University, Beijing 102206, China

**Keywords:** ceramic membranes, solid-state sintering, hydrophobic modification, coal fly ash, pore size, membrane distillation

## Abstract

Membrane distillation is an emerging separation technology with a high separation factor in water desalination. Ceramic membranes are increasingly used in membrane distillation because of high thermal and chemical stabilities. Coal fly ash is a promising ceramic membrane material with low thermal conductivity. In this study, three hydrophobic coal-fly-ash-based ceramic membranes were prepared for saline water desalination. The performances of different membranes in membrane distillation were compared. The effects of membrane pore size on permeate flux and salt rejection were researched. The coal-fly-ash-based membrane showed both a higher permeate flux and a higher salt rejection than the alumina membrane. As a result, using coal fly ash as the material for membrane fabrication can effectively increase the performance when applied to MD. Increasing the membrane pore size improved the permeate flux, but reduced the salt rejection. When the mean pore size increased from 0.15 μm to 1.57 μm, the water flux rose from 5.15 L·m^−2^·h^−1^ to 19.72 L·m^−2^·h^−1^, but the initial salt rejection was reduced from 99.95% to 99.87%. The hydrophobic coal-fly-ash-based membrane with a mean pore size of 0.18 μm exhibited a water flux of 9.54 L·m^−2^·h^−1^ and a salt rejection of higher than 98.36% in membrane distillation.

## 1. Introduction

Freshwater scarcity is perceived as a sobering global issue. Half a billion people suffer from severe water scarcity all year round worldwide [1,2]. It is imperative to develop economical and effective desalination technologies to obtain freshwater. In recent decades, numerous burgeoning and promising water purification technologies have exhibited efficiency and low cost, such as capacitive deionization (CDI) [3], the advance oxidation process (AOPs) [4], and so on. Membrane distillation (MD), which is an emerging membrane separation technology combining membrane separation with a conventional distillation process, is receiving increasing attention.

In an MD process, the hydrophobic membrane serves as a physical barrier to separate the salty feed liquid and the condensed water. Water vapors from the hot feed liquid permeate through the membrane to be condensed under the drive of the transmembrane partial pressure difference resulting from the temperature difference [5]. Compared with conventional thermal distillation, MD as a thermal-driven process can operate at a much lower temperature. Thus, the MD process can utilize solar energy, geothermal heat and other alternative low-grade energy sources [6,7]. Moreover, the operating pressure in the MD process is much lower than that in pressure-driven processes such as reverse osmosis and nanofiltration [8]. Furthermore, MD is theoretically capable of realizing 100% non-volatile solute rejection and the high salinity of feed liquid does not greatly influence the performance of the MD process [9,10].

MD can be classified into four modes, based on how the vapor pressure gradient is formed: direct contact membrane distillation (DCMD), air gap membrane distillation (AGMD), sweeping gas membrane distillation (SGMD), and vacuum membrane distillation (VMD) [11]. In accordance with their corresponding advantages and the properties of the feed liquid, the most appropriate MD mode is chosen for separation. Among these modes, VMD will increase the risk of pore wetting, which is significant for membrane performance [12]. Moreover, because of the higher energy requirements, VMD is more expensive energetically, especially when the operating temperature is over 75 °C [13]. Nevertheless, compared to other modes, the advantages of lower heat and mass transfer resistance as well as the higher flux and rejection performance of VMD make it widely studied.

Hydrophobic polymeric membranes including polyvinylidene fluoride (PVDF), polytetrafluoroethylene (PTFE), polypropylene (PP), and polyethylene (PE) are widely studied in the application of MD. However, for the applications in MD, a better chemical and thermal stability of membranes is required, in some cases involving high temperature and extreme pH environment [14]. Ceramic membranes possess the outstanding properties of high thermal and chemical stabilities and eminent mechanical strength and high flux, thus exhibiting promising potential in wastewater treatment via the MD process [15]. Therefore, applying the hydrophobic ceramic membranes to the MD process is attracting more and more interest from science researchers, due to these advantages. However, ceramic membranes are inherently hydrophilic because of the presence of hydroxyl groups (-OH) on the membrane surface. In order to realize the successful application of the ceramic membranes in MD and achieve the good effect of desalination of wastewater, hydrophobic modification on the membrane surface is an essential prerequisite [16]. To address this issue, grafting the hydrophobic organic compound is an effective and frequently-used method [17,18].

The first report on the application of the hydrophobic ceramic membrane in MD was submitted by Larbot et al. [19] in 2004. The alumina and zirconia membranes were grafted with fluoroalkylsilanes before being applied to MD, and obtained an excellent salt rejection of 100% with flux ranging from 0.5 to 8.43 L·m^−2^·h^−1^. Kujawa et al. [20] prepared a hydrophobic titania ceramic membrane and then applied it to the MD process in NaCl solutions in 2014. A salt retention coefficient of almost 100% was reached and the flux was in the range of 231–3692 g·m^−2^·h^−1^. Later, in 2016, Kujawa et al. [21] studied the influence of silanes structure and grafting time on the physicochemistry of the titania membrane surfaces. The fabricated membranes were then applied to MD and showed excellent selective property (~99% of salt rejection) and good transport property (permeate flux in the range of 0.5–4.5 kg·m^−2^·h^−1^ for 5 kD membranes). In the same year, Kujawski et al. [22] used the liquid entry pressure (LEP) as a measure of the hydrophobization efficiency and investigated the correlation of LEP and diverse parameters during the membrane preparation process, including type of molecule, concentration of FAS solution, type of solvent and duration of grafting. Moreover, it was found that the pore size of the hydrophobic membrane strongly affected the selectivity of membranes regarding the water–organic mixtures.

Researchers have focused a lot on the enhancement of the thermal efficiency and permeate flux in the MD process. The thermal efficiency can be defined as the ratio of vaporization latent heat to the total heat lost from the feed liquid, which includes the latent heat and the conducted heat through the membrane. In the MD process, a high temperature gradient between the two sides of the membrane is maintained to obtain high vapor flux, which contributes to high conductive-heat loss across the membrane. Thus, the massive loss of the vaporization heat and conductive heat leads to the low thermal efficiency of the MD system of only 20–30% [23]. Alobaidani et al. [24] indicated that increasing the thickness of the membrane, feed flow rate and feed temperature can enhance the thermal efficiency, but increasing the concentration of feed liquid will decrease the thermal efficiency.

In an MD process, thermal conductivity of the membrane plays an important role. Lower thermal conductivity exhibits a higher thermal resistance, leading to the decreased heat transfer through the membrane and making it easier to establish a higher transmembrane temperature difference on both side of the membrane, consequently obtaining a higher water flux [25,26]. Thus, it is indispensable to apply a lower thermal-conductivity ceramic membrane for a better performance in the MD process. At present, hydrophobic ceramic membranes used in membrane distillation are mainly derived from Al_2_O_3_. It has been proved that the thermal conductivity of a coal fly ash (CFA)-based ceramic membrane is lower than that of an alumina ceramic membrane [27], which means the CFA-based ceramic membrane is promising for better performances in MD than the alumina membrane. However, the use of coal-fly-ash-based ceramic membranes in membrane distillation is rarely reported. Moreover, using CFA as the raw materials of ceramic membranes can not only reduce the improper disposal of huge quantities of CFA, but also decrease the fabrication cost of membranes [28]. Thus, comparing the performances of the CFA-based ceramic membrane and the alumina ceramic membrane in the MD process is the focus of this work.

In this study, three hydrophobic CFA-based ceramic membranes and one hydrophobic alumina ceramic membrane were prepared via solid-state sintering and the immersion method. The performance of the prepared membranes in the VMD test is experimentally compared. In addition, the effect of pore size on the performance of membranes in the VMD test is also studied.

## 2. Experimental Procedures

### 2.1. Materials

Polyvinyl Alcohol (PVA) was provided by Shandong Usolf Chemical Technology Co., Ltd. (Linyi, China) Sodium hexametaphosphate (AR), sodium chloride (NaCl, 99.9%), 1H,1H,2H,2H-Perfluorodecyltrichlorosilane (96%), ethanol (AR) and alumina nanoparticles (Al_2_O_3_, 99.9%) with a mean particle size of 200 nm were purchased from Shanghai Aladdin Biochemical Technology Co., Ltd. (Shanghai, China) Alumina powders (Al_2_O_3_) with a mean particle size of 1 μm were obtained from Zhejiang Lixie Instrument Equipment Co., Ltd. (Yiwu, China)

The CFA-based ceramic support used to fabricate the membrane was prepared using the extrusion method from the previous work [29]. The commercial alumina membrane was supplied by Jiuwu Hi-Tech Co., Ltd. (Nanjing, China) The internal and external diameter of these membranes were 8 and 12 mm, respectively, while the lengths were cut to 0.5 m. The salt solution was prepared by mixing sodium chloride with deionized water to a NaCl concentration of 10,000 ppm for desalination in VMD.

### 2.2. Preparation of CFA-Based Ceramic Membranes

The CFA-based ceramic support without any processing is named M1, while the commercial Al_2_O_3_ ceramic membrane purchased on the market is named M4.

Manufacturing of the asymmetric membrane was achieved via the solid-state sintering process based on the ceramic support. The Al_2_O_3_ particle suspension was formulated by dissolving PVA and sodium hexametaphosphate into deionized water under magnetic stirring and then adding alumina nanoparticles of 1 μm in. The weight ratio of PVA: sodium hexametaphosphate: alumina: H_2_O was 1:1:20:200. The sodium hexametaphosphate of the suspension acted as a dispersant while the PVA functioned as a binder. The first alumina separation layer was elaborated through the slip casting method involving the suspension on the ceramic support. The modified ceramic support was then aligned using a straightening machine (XZJ-25/1000, Henan Xinlong Machinery Factory, Xuchang, China) and dried for 24 h at room temperature, which was then followed by a calcination step from ambient temperature to 1150 °C using a preprogrammed automatic-control electric furnace (LYL-17LB, Luoyang Liyu kiln Co., Ltd., Luoyang, China). It should be noticed that a slow temperature-increase rate of 1–2 °C/min in the furnace was set to obtain crack-free membranes. The fabricated CFA-based membrane was named M2.

The preparation of a second separation layer was carried out on the basis of M2, and the operation procedure was same as that of the fabrication of M2. The only difference was that the alumina nanoparticles of 1 μm in the preparation step of the suspension were changed into alumina powder with a particle size of 200 nm, and the CFA-based membrane obtained was named M3. The details of these membranes are listed in Table 1. The microstructure parameters of the four membranes were measured before the hydrophobic modification.

### 2.3. Preparation of Hydrophobic Ceramic Membranes

**Figure 1:**
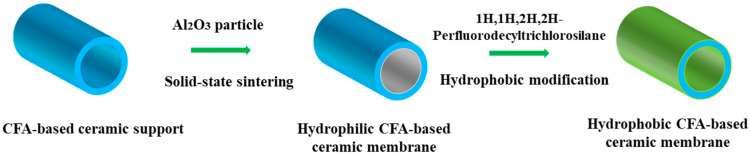
Schematic for the fabrication procedure of the CFA-based ceramic membranes.

The fabricated CFA-based ceramic membranes exhibit hydrophilic character because of the existence of hydroxyl groups on the membrane surface, and thus it is necessary to apply hydrophobic modification to the hydrophilic membranes for application to MD. In this work, surface grafting modification was applied on M1, M2, M3 and M4 to elaborate hydrophobic membranes via the immersion method from the previous work [30]. The hydrophilic membranes were successively ultrasonically cleaned for 10 min with deionized water and ethanol for the surface pretreatment before the desiccation treatment for 6 h. The pretreated surface membranes were then immersed in pre-prepared ethanol solution of 1H,1H,2H,2H-Perfluorodecyltrichlorosilane with a concentration of 0.2 mol/L for 12 h. After this, the modified membranes were rinsed using deionized water and dried for 12 h and the hydrophobic membranes were obtained. The elaborated hydrophobic membranes were named M5, M6, M7 and M8. The above-mentioned fabrication procedure of the membranes is presented simply in Figure 1.

### 2.4. Characterization

The cross-sectional and inner-surface morphology of the fabricated membranes were determined using scanning electron microscopy (SEM, Quanta 400 FEG, FEI, Hillsboro, OR, America). The mercury intrusion method was used to determine the pore size distribution of membranes using an automatic mercury porosimeter (Autopore IV 9520, Micromeritics Instruments Corporation, Norcross, GA, USA). In order to confirm the performance of hydrophobic modification, the static contact angles of the hydrophobic membranes were measured using the sessile drop method with a contact angle analyzer (DropMeter A-100P, MAIST Vision Inspection & Measurement Co., Ltd., Shenzhen, China). Drops of deionized water with a volume of 2 μL were placed on the internal surface of the hydrophobic membranes and the left and right contact angles were immediately measured, using the analyzer.

The pure water flux of the hydrophilic membranes (M1, M2, M3 and M4) was measured in the laboratory using a test bench, as described in the previous work [29]. The permeate flow rate of pure water is calculated by Equation (1).

*J* = *V*/(*A*·Δ*t*) (1)

where *V* is the volume of permeated water (L), Δ*t* is the operating time (h), and *A* is the inner surface area of the membrane (m^2^).

**Figure 2:**
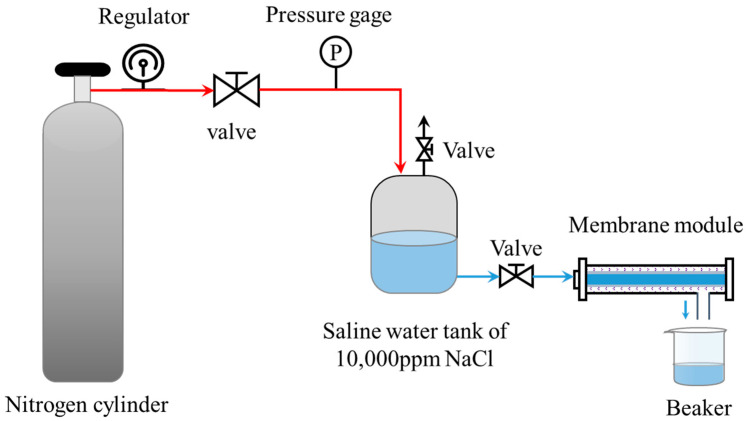
Schematic of the setup for LEP measurement on the hydrophobic membranes.

The liquid entry pressure (LEP) of the hydrophobic membranes (M5, M6, M7 and M8) was determined by a test bench using the prepared NaCl solution with concentration of 10,000 ppm. The setup for LEP measurement on the hydrophobic membranes was self-built, as shown in Figure 2. The NaCl solution was pressed into the membrane module by the nitrogen from the cylinder. The regulator was used for adjusting pressure and the pressure gage was used to monitor pressure. The pressure was increased by 1 kPa every time and each pressure was sustained for at least 5 min. Pressure was continuously increased until the solution was collected in the beaker, and the pressure was recorded as the LEP of the membrane at this time.

### 2.5. Membrane Distillation Test Using Hydrophobic Ceramic Membranes

**Figure 3:**
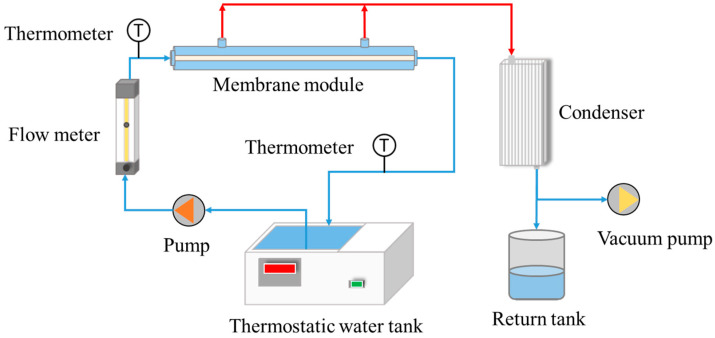
Schematic of the vacuum membrane distillation test.

The fabricated hydrophobic ceramic membranes (M5, M6, M7 and M8) are used for saline water desalination in the MD test in order to study their different performances. The experimental apparatus presented in Figure 3 is used for water desalination with the membranes via vacuum membrane distillation. The NaCl solution, that is the feed liquid, is pre-heated in the thermostatic water tank before being sent into the membrane module and flowing up through the inner side of the membrane. The temperature, flow rate, and pressure of the feed liquid are measured by a thermometer, a flowmeter, and a manometer, respectively. At the outside of the membrane, the water vapor permeates through the membrane under the driving force of heat and differential pressure and is then condensed into liquid water in the condenser and collected in the tank. The salt rejection and permeate flux are the two important parameters for evaluating the hydrophobic membrane performance in the membrane distillation test [31]. The weight of the collected water is measured by an electronic balance, while the Na^+^ concentration of the feed liquid and collected water is determined by measuring their conductivities via a conductivity meter. Therefore, the permeate flux *J* (L·m^−2^·h^−1^) can be calculated by Equation (1).

The salt rejection of the membrane *R* can be obtained from Equation (2).

*R* = (*C_f_* − *C_p_*)/*C_f_* × 100% (2)

where *C_f_* and *C_p_* stand for the Na^+^ concentration of the feed liquid and the collected water, respectively.

## 3. Results and Discussion

### 3.1. Characterization of Fabricated Membranes

#### 3.1.1. Morphology and Element Composition

**Figure 4:**
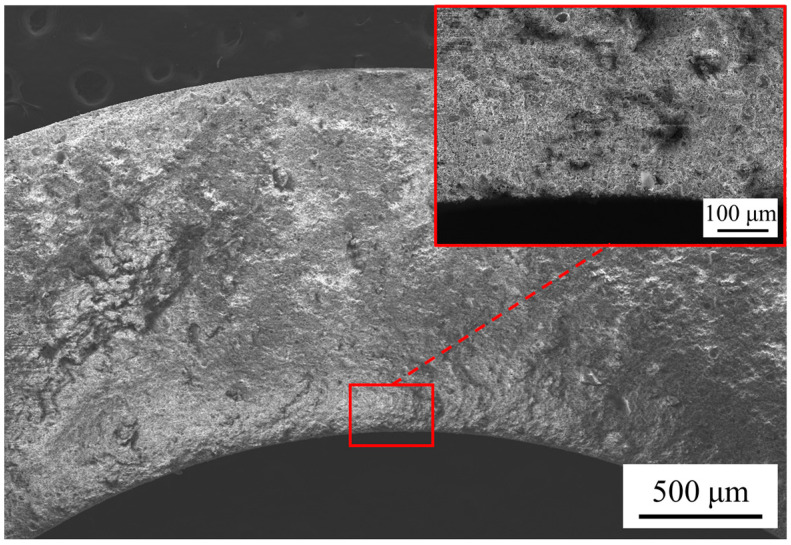
The site chosen on the sample for cross-sectional morphology by SEM.

**Figure 5:**
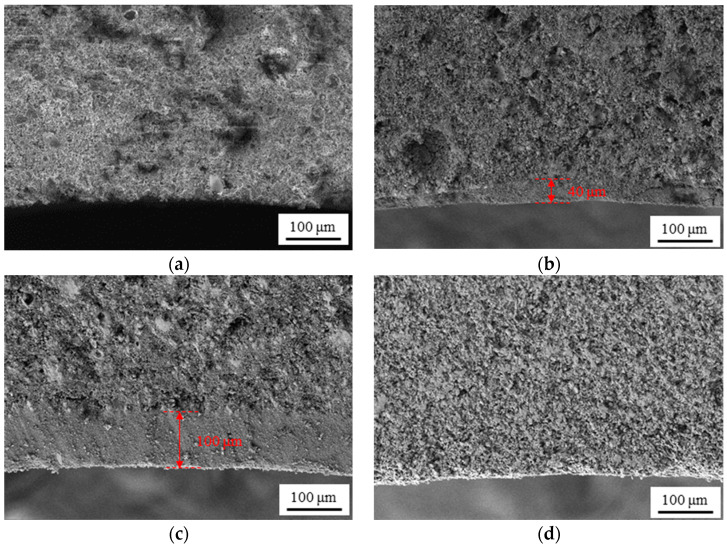
Cross-sectional SEM images of the four different membranes. (**a**) M1; (**b**) M2; (**c**) M3; (**d**) M4.

**Figure 6:**
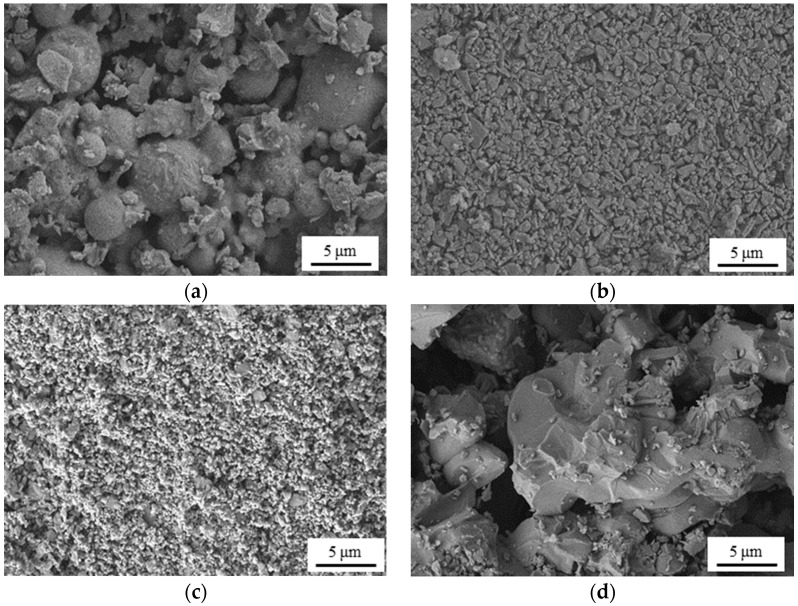
Inner-surface SEM images of the four different membranes. (**a**) M1; (**b**) M2; (**c**) M3; (**d**) M4.

Scanning electron microscopy (SEM) is used to observe the cross-sectional and surface morphology of the membrane in numerous studies on membrane preparation [32,33,34]. In this study, the cross-sectional and inner-surface morphology of the fabricated membranes are determined by SEM. The fabricated membranes were broken into tiny fragments of less than 10 mm in length and width to prepare samples for SEM. The samples were sprayed with platinum for 1 min before the SEM observation. Using M1 as an example, Figure 4 shows the site chosen on the sample when observing the cross-sectional morphology of the membrane. The cross-sectional and inner-surface morphology of the fabricated membranes are shown in Figure 5 and Figure 6. It can be seen from Figure 5d that a thin layer with a thickness of 40 μm is coated on the support of M2, while the layer is much thicker on the support of M3, the thickness of which reaches 100 μm. From Figure 5c, the images present a complete and uniform cross-section of the membranes, which is consistent with the fact that M1 and M4 are the ceramic supports without any coating process of separation layers.

Figure 6 shows the inner-surface morphology of the four hydrophilic membranes (M1, M2, M3 and M4). No obvious cracks or other defects are observed from Figure 6b,c, demonstrating the successful coating of the separation layer on M1 and M2. Moreover, a phenomenon occurs wherein the surface of the separation layers of M2 and M3 are dense and uniform without obvious particle aggregation, which can be explained by the addition of dispersant in the suspension, thus applying the dispersion effect on the Al_2_O_3_ particles. Comparing Figure 6a–c, it can be seen that the inner surface is clearly becoming denser through the coating process of the first and second separation layer, indicating the shrinkage in pore size.

**Figure 7:**
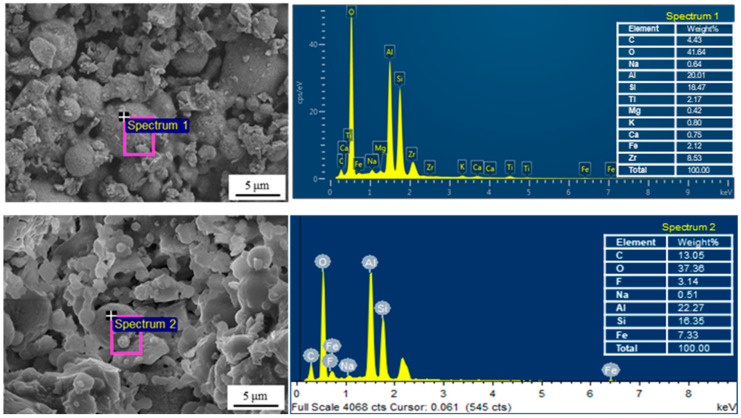
EDS spectrum of M1 (Spectrum 1) and M5 (Spectrum 2).

The EDS elemental analysis is conducted on the surface of the hydrophilic CFA-based membrane M1 and the hydrophobic CFA-based membrane M5, and the result is shown in Figure 7. As the hydrophilic CFA-based ceramic support is composed mainly of Al, Si and O, and there is an element F in CFA [29], the high absorption-peak intensity of Al, Si and O in the Spectrum 1 indicates the existence of CFA, and the absorption-peak intensity of element F in Spectrum 2 proves that 1H,1H,2H,2H-Perfluorodecyltrichlorosilane has been successfully grafted onto the surface of the hydrophilic CFA-based membrane.

#### 3.1.2. Pore Size Distribution

**Figure 8:**
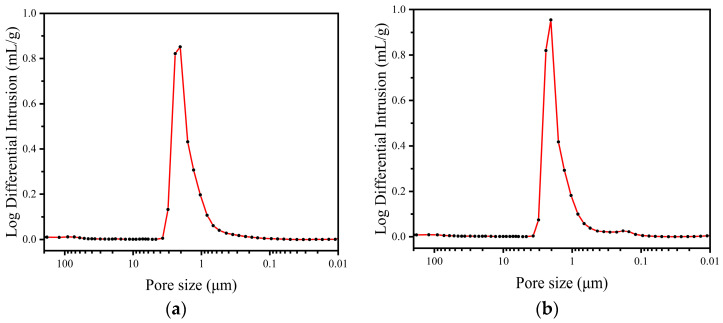

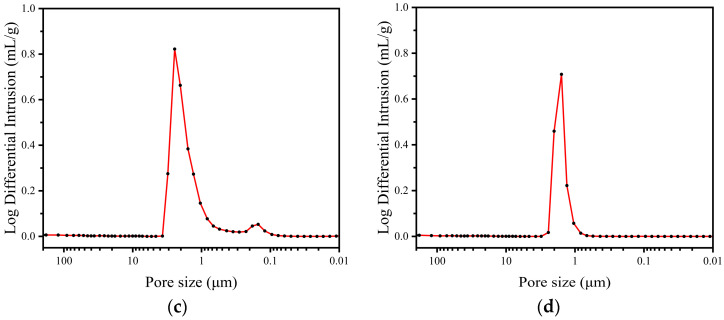
Pore size distribution of the four different membranes via mercury intrusion method. (**a**) M1; (**b**) M2; (**c**) M3; (**d**) M4.

The mercury intrusion method is used to determine the pore size distribution of the fabricated membranes. The pore size distribution of M1, M2, M3 and M4 is shown in Figure 8. It can be seen from Figure 8b,c that both curves present two peaks. For M2, the first peak occurs at a pore diameter of 0.83–3.08 μm, and the mean pore diameter is 2.04 μm, which represents the pore size distribution of the support. The second peak occurs at 0.15–0.28 μm, and the mean pore diameter is 0.18 μm, representing the pore size distribution of the separation layer. For M3, the two peaks occur at a pore diameter of 0.83–3.08 μm and 0.12–0.28 μm, while the mean pore diameters are 2.05 μm and 0.15 μm, representing the pore size distribution of the support and the separation layer, respectively. The pore volume fraction of the support in M2 is 94.13%, and that of the separation layer is 5.87%, while the pore volume fraction of the support in M3 is 92.75%, and that of the separation layer is 7.25%. As the preparation process of M3 applies the second slip casting on M2, the difference in pore size distribution between M2 and M3 indicates that the second slip casting successfully shrinks the average pore size of the separation layer. The experimental results reported in the literatures [35,36] also indicate that the second slip casting shrinks the mean pore size of the separation layer. In addition, because of the increasing thickness of the separation layer of M3, the pore volume fraction of the separation layer has increased. Both curves in Figure 8a,d present the only peak, which is in accordance with the fact that M1 and M4 are the ceramic supports without any separation layers. The pore size of M1 and M4 are concentrated in 0.83–3.09 μm and 1.04–2.01 μm, and the mean pore diameter are 2.05 μm and 1.57 μm, respectively.

From the results above, the pore size distribution of the ceramic support of M1, M2 and M3 is basically the same, indicating that the coating of the separation layer has a negligible effect on the characteristics of the ceramic support but forms an extra layer on the surface of the support. Moreover, the mean pore diameter of the separation layer of M3 is smaller than that of the separation layer of M2, which is tinier than that of M1. The relationship of their mean pore diameters explains the successful shrinkage in the pore size of the separation layer after the coating process, which is the data analysis of the SEM images, as shown in Figure 6.

#### 3.1.3. Pure Water Flux

**Figure 9:**
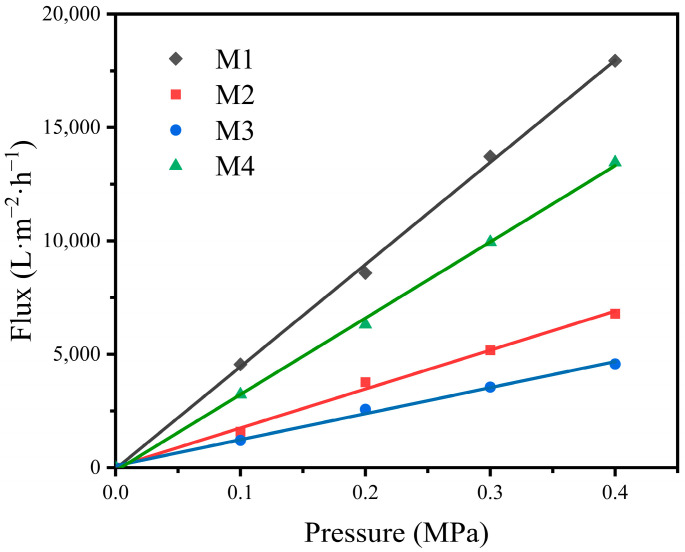
Pure water flux of the four different hydrophilic membranes.

Pure water flux is an important evaluation index of the fabricated hydrophilic membrane performance. The pure water flux through the four different hydrophilic membranes was calculated by the permeate flow rate during the pure water flux measurement and the results are shown in Figure 9. It can be seen from Figure 9 that the slopes of the permeate flow rate versus the transmembrane pressure plots correspond to the pure water flux of the membranes, which is an evaluation index of membrane performance. The pure water flux of M1, M2, M3, M4 is 4550, 1584, 1209, 3225 L·m^−2^·h^−1^·bar^−1^, respectively, which shows a positive correlation with the pore size distribution of the four different membranes. According to the Hagen–Poiseuille equation [37], the pore size is one of the main indexes affecting the pure water flux, and thus the increase in the pore size leads to the rising trend of the pure water flux, which is consistent with the conclusions drawn from Figure 9.

#### 3.1.4. Wettability

**Figure 10:**
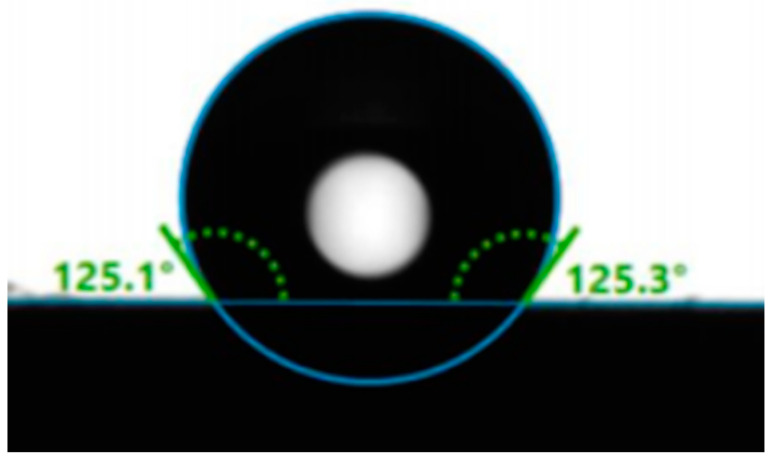
Water contact angle of hydrophobic membrane (M7) after modification.

In order to quantitatively analyze the inner surface wettability of the four modified ceramic membranes (M5, M6, M7, M8) used in the VMD process, water contact angles (CA) were test. The water contact angle of M7 is shown in Figure 10. After hydrophobic modification, deionized water formed a stable contact angle of an average of 125.2° on the membrane inner surface. Furthermore, besides M7, water contact angles of the other three hydrophobic membranes (M5, M6, M8) are also shown in Table 2. The results indicate that the water contact angles of the modified membranes are similar, and thus the wettability difference among the four hydrophobic membranes has little effect on their application to the VMD process. Moreover, for the four hydrophilic membranes (M1, M2, M3, M4), water droplets were immediately absorbed when dripped onto the membrane surfaces and the static contact angles of the hydrophilic membranes were 0°. The values of the contact angle for the hydrophobic membranes were in the range of 123–128°, indicating that the hydrophobic modification had successfully changed the hydrophilicity into hydrophobicity for the fabricated membranes.

#### 3.1.5. Liquid Entry Pressure (LEP)

The LEP measurements and property of the four hydrophobic membranes are shown in Table 3. In order to make sure that the membrane wetting phenomenon does not occur in the membrane distillation process, the minimum value of LEP should be selected, and the pressure difference between the two sides of the membranes should be set below the minimum LEP value. Thus, the pressure difference is set to 0.06 MPa and the pressure in the permeate side is maintained at 0.04 MPa in the MD test.

### 3.2. Vacuum Membrane Distillation Test

#### 3.2.1. VMD Performance at Different Feed Flow Rates

**Figure 11:**
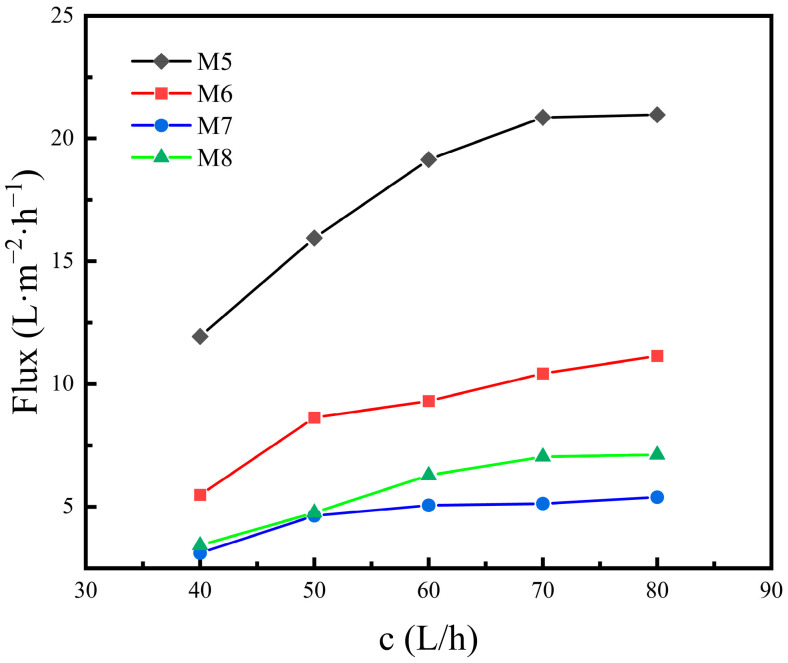
Water flux versus feed flow rate of the four different membranes in the VMD test.

**Figure 12:**
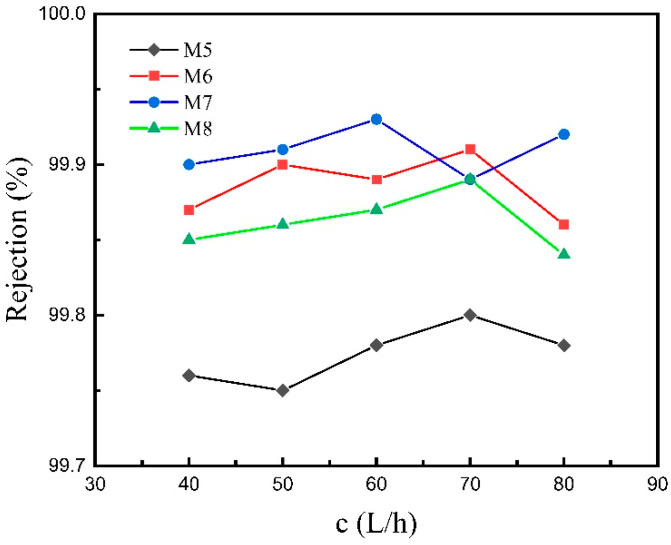
Salt rejection versus feed flow rate of the four different membranes in the VMD test.

The fabricated hydrophobic membranes are applied to the VMD test to evaluate their performance. The tests are carried out by dissolving sodium chloride in DI water and using the hydrophobic membranes to desalinate the saline water. Two main parameters, the water flux and salt rejection, are evaluated to determine the performance of the membranes. The influence of the feed flow rate on the water flux and salt rejection is studied at the constant feed temperature of 55 °C. The apparatus for the MD process operates steadily for at least 1 h before the MD test begins. Subsequently, for each condition changed, the permeated water is collected for 0.5 h and the permeate flux and salt rejection is measured. The experimental results are plotted into curves in Figure 11 and Figure 12. As is shown in Figure 11, it can be concluded that, when the feed flow rate is higher, the rising trend of water flux decreases gradually, that is, the influence of flow rate on the water flux decreases gradually. For M5, the water flux increases from 11.94 to 19.14 L·m^−2^·h^−1^ when the feed flow rate rises from 40 to 60 L/h. However, when the feed flow rate continuously rises to 80 L/h, the increase in the water flux is limited, and it increases to 20.97 L·m^−2^·h^−1^ only. The reason for the changing law is that there are two phenomena, concentration polarization and temperature polarization, in the membrane distillation process. Concentration polarization means that in the process of membrane distillation, non-volatile Na^+^ and Cl^−^ will converge on one side of the membrane to form a high concentration of ion layer and reduce the partial pressure of the water vapor. Temperature polarization refers to the formation of a certain thickness of the temperature boundary layer on the surface of the membrane, resulting in the fact that the temperature difference across the two sides of the membrane is lower than the temperature difference of the fluid body on both sides of the membrane, and causing the reduction in the driving force of mass transfer formed by the temperature difference.

When the flow rate of the feed liquid rises, the disturbance of the fluid is strengthened, which makes the boundary layer on the membrane surface thinner; the temperature polarization and concentration polarization weaken, and the saturated vapor pressure of the feed liquid increases, which increases the driving force of mass transfer on the hot side of the membrane, thus leading to the increase in the water flux. In addition, with the acceleration of the flow rate, the residence time of the feed liquid in the membrane module is shorter, which reduces the temperature drop of the feed liquid, and the operation temperature is increased during the membrane distillation process, which further increases the driving force of the mass transfer.

From Equation (3), the Reynolds number can be calculated regarding the feed flow rate. The calculated Reynolds number is 2075.4 when the feed flow rate is 50 L/h and the number is 2502.5 when the flow rate is 60 L/h. As the feed flow rate continues to rise from 50 L/h and the critical Reynolds number of 2320 is exceeded, the flow inside the membrane changes from laminar to turbulent; thus, the effect of the increase in flow rate on improving the thermal efficiency of the boundary layer is limited, and the increase in the driving force of the mass transfer is weakened, so the increasing trend of the water flux gradually decreases.

*Re* = *cl*/*ν* (3)

where *c* is the feed flow rate, *l* is the inner diameter of the membrane, and *ν* is the kinematic viscosity of the feed liquid.

From the curves demonstrated in Figure 12, it can be seen that as the feed flow rate increases, the curves of the salt rejection fluctuate slightly, but the overall curve tendency maintains a nearly horizontal trend; that is, the change in the flow rate has little effect on the salt rejection. For M5, the salt rejection fluctuates in the range of 99.75% to 99.80% when the feed flow rate rises from 40 to 80 L/h, which exhibits a slight change in salt rejection. The changing law of salt rejection with the feed flow rate indicates that the temperature polarization and concentration polarization have little effect on the salt rejection during the MD process.

#### 3.2.2. VMD Performance at Different Feed Temperatures

**Figure 13:**
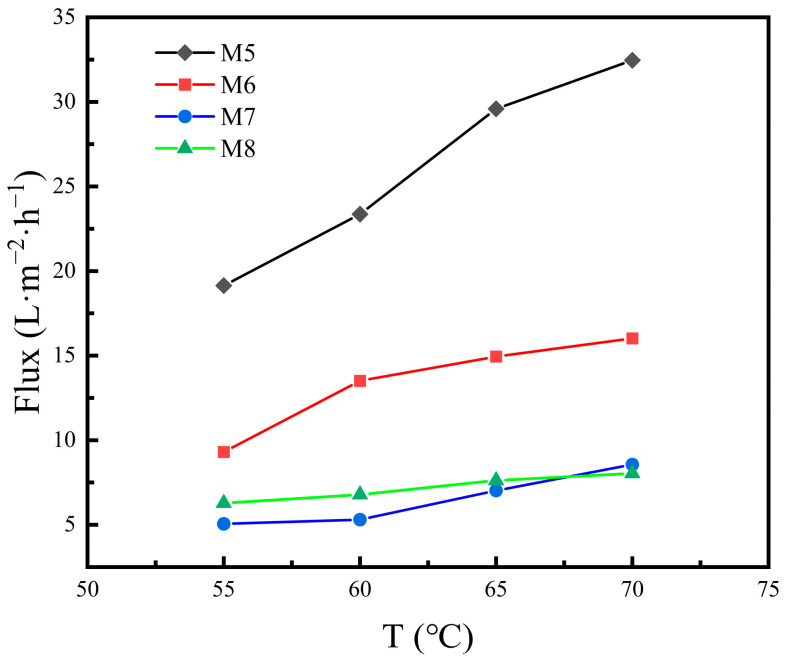
Water flux versus feed temperature of the four different membranes in the VMD test.

**Figure 14:**
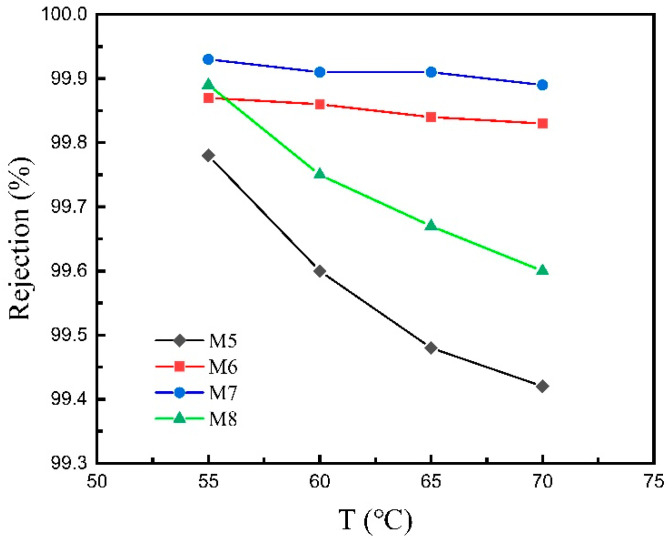
Salt rejection versus feed temperature of the four different membranes in the VMD test.

The change in water flux and salt rejection with feed temperature of the four different hydrophobic membranes at the constant feed flow rate of 60 L/h is shown in Figure 13 and Figure 14. As the temperature increases, the water flux of the four membranes increases, because the pressure difference caused by the temperature difference on both sides of the membrane is the driving force of the water vapor transported across the membrane. Therefore, the higher the temperature, the greater the driving force, and the greater the water flux of the membrane. For M5, the water flux increases from 19.14 to 32.46 L·m^−2^·h^−1^ when the feed temperature rises from 55 to 70 °C. The improvement in water flux is enormous, which indicates that raising the feed temperature can strongly enhance the water flux during the MD process. However, with the increase in temperature, the salt rejection of the four membranes decreases, because the higher the temperature, the worse the stability of the hydrophobic layer, and the higher the temperature, the more easily the hydrophobic layer is destroyed, resulting in the decrease in hydrophobicity; a small amount of salt solution may permeate the membrane, resulting in the doping of impurity ions in the water recovered by the permeate side, so the salt rejection decreases. For M5 and M8, the tendency of salt rejection to decrease with feed temperature is similarly sharp, as when the feed temperature rises from 55 to 70 °C the salt rejection decreases from 99.78% to 99.42% for M5 and it decreases from 99.89% to 99.6% for M8. However, for M6 and M7, the overall curve tendency maintains a nearly horizontal trend, indicating that the influence of feed temperature is relatively slight on the salt rejection. The different decline tendency of salt rejection among M5, M8 and M6, M7 is likely due to the difference in mean pore size, which is explained by the fact that the smaller mean pore size may alleviate the decline in salt rejection affected by the rise in feed temperature.

From Figure 11, Figure 12, Figure 13 and Figure 14, it can be concluded that, under the same feed liquid temperature and flow rate, the water flux of M5 is the highest, and that of M6 and M7 decreases successively. However, the relationship of the salt rejection is the opposite. The phenomenon is related to the mean pore size. M7, with two separation layers, exhibits a mean pore size of 0.15 μm. M6 possesses a mean pore size of 0.18 μm, which is the mean pore size of the only separation layer. While M5 is the ceramic support without any separation layer, so its mean pore size is the mean pore size of the support, which is 2.05 μm. Thus, the conclusion can be drawn that the water flux shows a positive correlation with the mean pore size. However, the salt rejection goes down with the rise in the mean pore size. The opposite variation tendency of the water flux and the salt rejection coincides with the trade-off effect.

From the pore size distribution shown in Figure 8, it can be seen that the mean pore size of M8 is 1.57 μm, which is the mean pore size of the support. From Figure 11 and Figure 13, it can be found that when the feed liquid temperature and flow rate is the same, the water flux of M8 is between that of M6 and M7. However, the mean pore size of M8 is larger than that of M6, which is even larger than that of M7. According to the conclusion above, the water flux of M8 should theoretically be higher than that of M6 and M7. The water flux of M8 is experimentally lower than the result of the theory analysis, which is attributed to the material difference of M8 and the other membranes. Moreover, there are no separation layers of M5 and M8, and the mean pore size of these are both over 1 μm. However, the water flux of M5 is much higher than that of M8, which further explains the fact that the great difference in water flux is attributed to the material difference. Because the thermal conductivity of the CFA-based ceramic membrane is lower than that of the alumina ceramic membrane, it is easier to establish a large temperature gradient on both sides of the membrane, and the pressure difference generated by the temperature difference on both sides of the membrane is the driving force for promoting the water vapor transport across the membrane. Therefore, the water flux in the process of membrane distillation is higher under the higher temperature gradient, which gives rise to the fact that M8 exhibits a lower water flux and even possesses a larger mean pore size compared to the other membranes.

#### 3.2.3. The Stability of Hydrophobic Ceramic Membranes in the VMD Test

**Figure 15:**
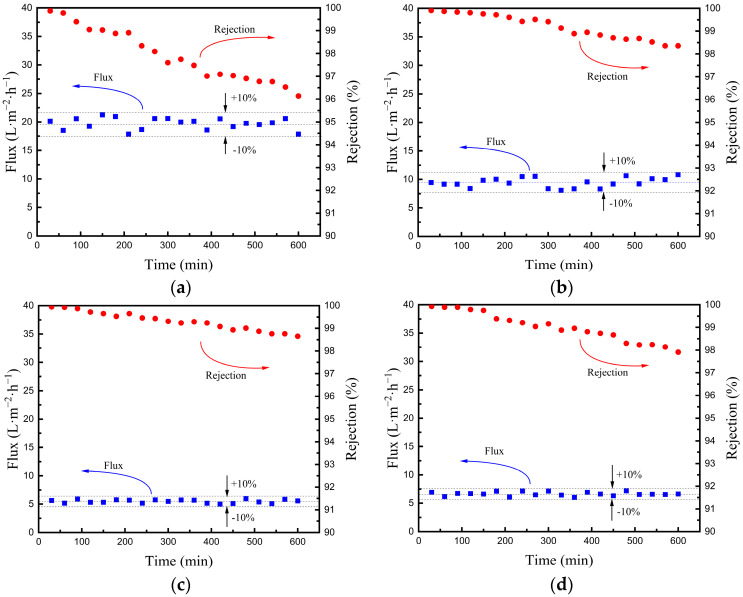
The changes in water flux and salt rejection over time in the VMD test. (**a**) M5; (**b**) M6; (**c**) M7; (**d**) M8.

In practical applications, not only water flux and salt rejection but also a continuous operating period is critical for a membrane to be applied in waste water or saline water desalination. The continuous operating period of the four hydrophobic membrane is evaluated by performing the VMD experiment for several hours when the feed flow rate is sustained at 60 L/h and the feed temperature is maintained at 55 °C. Each membrane is individually applied to the VMD test and steadily operated for 10 h. The changes in water flux and salt rejection over time of the four different hydrophobic membranes are shown in Figure 15. The average water flux of M5, M6, M7 and M8 is 19.72, 9.54, 5.15, and 6.62 L·m^−2^·h^−1^, respectively, and the fluctuation of the water flux is no more than ±10%. The initial salt rejection of M5, M6, M7 and M8 is 99.89%, 99.91%, 99.95%, and 99.93%, respectively, while the final salt rejection of M5, M6, M7 and M8 is 96.23%, 98.36%, 98.72%, and 97.91%, respectively.

From the data obtained, it can be concluded that as the mean pore size of CFA-based membranes (M5, M6 and M7) decreases, the average water flux reduces, but both the initial and final salt rejection rises. In addition, the average water flux of M8 is higher than that of M7 but lower than M6, which is in keeping with the results obtained from previous experiments. Another conclusion that can be drawn is that the water flux fluctuates slightly and the salt rejection declines mildly after operating for 10 h, which indicates that the hydrophobicity of the fabricated hydrophobic membranes is stable in the VMD process.

Moreover, among the three hydrophobic CFA-based membranes (M5, M6, and M7), the salt rejection of M5 declines relatively rapidly compared with the other membranes, although its water flux is the highest. To obtain pure water from the MD process, M5 may not be the best choice in the application of water desalination. However, the initial salt rejection of M6 (99.91%) is not much lower than that of M7 (99.95%), and the decreasing tendency of the salt rejection is similar to that of M7, which is relatively small compared to M5, although the average water flux is nearly double that of M7, reaching 9.54 L·m^−2^·h^−1^. Therefore, considering the water flux, the salt rejection and the stability, the CFA-based membrane M6 exhibits the best performance in the VMD process. The initial salt rejection of M6 is 99.91% and the final salt rejection is 98.36%, while the water flux is 9.54 L·m^−2^·h^−1^.

The performance of M6 in the MD process is comparable to that of membranes reported in the literature [26,38,39,40,41,42,43,44,45], as can be seen from Table 4. Using CFA as the material for membrane fabrication is highly promising because CFA is low-cost and the fabrication method is inexpensive, while the other membranes mentioned in Table 4 are fabricated from more expensive materials such as alumina, zirconia and titania. In this work, all of the four hydrophobic membranes exhibit high initial salt rejection of more than 99.8%, which is higher than the salt rejection in some of the literature [26,38,39,43,44,45], and close to that of other studies [40,41,42]. The CFA-based membrane M5 performs the highest water flux, of 19.72 L·m^−2^·h^−1^, which is higher than most of other membranes listed in Table 4. However, considering the water flux, the salt rejection and the stability, the CFA-based membrane M6 performs best in the VMD process among the four hydrophobic membranes. The water flux of M6 (9.54 L·m^−2^·h^−1^) is higher than the hydrophobic alumina membrane fabricated in this work and many other membranes listed in Table 4. For example, the water flux of M6 is higher than that in some of the literature [26,38,39,40,43], but lower than that of other studies [41,42,44,45]. However, the higher water flux presented in some of the literature [41,42,44,45] is due to membranes with a larger mean pore size. The results conform well with the conclusion mentioned above: the performance of the hydrophobic CFA-based membrane in the VMD process is better than that of the hydrophobic alumina membrane.

## 4. Conclusions

In this study, we elaborate three hydrophobic coal-fly-ash-based (CFA-based) ceramic membranes and a hydrophobic alumina ceramic membrane based on the extrusion and immersion methods. The performance of CFA-based ceramic membranes and commercial alumina ceramic membranes in the vacuum membrane distillation (VMD) test is comparatively studied. In addition, the effect of mean pore size on performance in the VMD test is also researched. Several conclusions can be drawn from the results:

(1) In the vacuum membrane distillation process, with the increase in the mean pore size, the water flux through the membranes rises, but the salt rejection reduces, which is consistent with the trade-off effect. When the mean pore size increases from 0.15 μm to 1.57 μm, the water flux rises from 5.15 L·m^−2^·h^−1^ to 19.72 L·m^−2^·h^−1^, but the initial salt rejection reduces from 99.95% to 99.87%.
(2) The performance of the CFA-based ceramic membrane in the VMD process is better than that of the alumina ceramic membrane. The alumina ceramic membrane possesses a larger mean pore size (1.57 μm) than that of the CFA-based membrane (0.18 μm), but exhibits a lower water flux during the VMD process of only 6.62 L·m^−2^·h^−1^, while the water flux of the CFA-based membrane reaches 9.54 L·m^−2^·h^−1^.
(3) Considering the water flux, the salt rejection and the stability, the CFA-based membrane with a mean pore size of 0.18 μm exhibits the best performance in the VMD process, with a water flux of 9.54 L·m^−2^·h^−1^ and a salt rejection of higher than 98.36%.
(4) The stability test of the four hydrophobic membranes in the VMD process is carried out. The results show that the water flux fluctuates slightly and the salt rejection declines mildly after operating for 10 h, which indicates that the hydrophobicity of the fabricated hydrophobic membranes is stable in the VMD process.

## Figures and Tables

**Table 1:** Characteristics of the four membranes.

Membranes	Support			Separation Layer	
	Material	Internal/External Diameter (mm)	Length (m)	Number of Layers	Thickness (μm)
M1	CFA	8/12 ± 0.23	0.5 ± 2.88 × 10^−3^	0	0
M2	CFA			1	40 ± 4.96
M3	CFA			2	100 ± 7.93
M4	Al_2_O_3_			0	0

**Table 2:** Water contact angles of the four hydrophobic membranes.

Membranes	CA (°, Left)	CA (°, Right)	CA (°, Average)
M5	123.0	123.2	123.1
M6	127.9	127.9	127.9
M7	125.1	125.3	125.2
M8	125.5	125.4	125.5

**Table 3:** The LEP measurements and property of the four hydrophobic membranes.

Membranes	Maximum Pore Size (×10^−6^ m)	LEP (MPa)
M5	2.12	0.066
M6	2.41	0.065
M7	2.46	0.062
M8	2.44	0.061

**Table 4:** Performance of several kinds of membranes in MD process for desalination.

Membrane	MD Type	Feed Solution	Pore Size (μm)	Water Flux (L·m^−2^·h^−1^)	Salt Rejection (%)	Ref.
Zirconia	VMD	NaCl (17,000 ppm)	0.05	7.5	>99	[38]
Titania	VMD	NaCl (17,000 ppm)	0.005	6.08	>99	[38]
Zeolite membrane	VMD	NaCl (35,000 ppm)	0.07	5.2	99	[26]
Red clay ceramic	VMD	NaCl (10,000 ppm)	0.035	6.39	>80	[39]
Zirconia	AGMD	NaCl (0.1 mol·L^−1^)	0.05 0.2	~6	~100	[40]
Alumina	VMD	NaCl (3.5 wt%)	0.4	29.3	99.9	[41]
Alumina	VMD	NaCl (1 wt%)	0.2	27.28	99.99	[42]
Alumina-silica	AGMD	Seawater	0.1	3.3–7.5	98.5–99	[43]
Alumina	DCMD	NaCl (2 wt%)	0.76	19.1	>99.5	[44]
Alumina	SGMD	NaCl (4 wt%)	~0.8	21	>99	[45]
CFA	VMD	NaCl (10,000 ppm)	0.18	9.54	98.36	This work

## Data Availability

The data presented in the study are available upon request from the corresponding authors.
